# Targeting glucocorticoid receptor signaling in platinum-resistant ovarian cancer: translational rationale and clinical advances following the ROSELLA trial

**DOI:** 10.3389/or.2026.1861081

**Published:** 2026-07-09

**Authors:** Wenxiang Wang, Zhanghuan Li, Junjie Zhao, Mengyuan Chang, Yafang Zhang, Xiangyi Shen, Junming Yue, John Schorge, Wenjing Zhang

**Affiliations:** 1 Department of Gynecologic Oncology, Xinxiang Central Hospital, The Fourth Affiliated Hospital of Henan Medical University, Xinxiang, Henan, China; 2 Department of Pathology, College of Medicine, University of Tennessee Health Science Center, Memphis, TN, United States; 3 Center for Cancer Research, University of Tennessee Health Science Center, Memphis, TN, United States; 4 Department of Obstetrics and Gynecology, University of Tennessee Health Science Center, Memphis, TN, United States

**Keywords:** clinical research, ovarian cancer, platinum resistance, ROSELLA trial, selective glucocorticoid receptor antagonist, targeted therapy

## Abstract

Platinum-resistant ovarian cancer (PROC) remains a major clinical challenge, with limited effective treatment options and poor survival outcomes. Aberrant activation of the glucocorticoid receptor (GR) signaling pathway promotes tumor progression, chemoresistance, and immune evasion, providing a strong rationale for therapeutic targeting. Selective GR antagonists (SGRAs) represent an emerging novel strategy aimed at blocking this pro-tumorigenic pathway while minimizing off-target effects. Preclinical studies demonstrated that GR blockade restores chemosensitivity, particularly to taxane-based therapies, and may modulate the tumor microenvironment. These findings have translated into clinical evaluation, culminating in the phase 3 ROSELLA trial, which reported that the addition of relacorilant, a SGRA, to nab-paclitaxel significantly improved progression-free and overall survival in patients with recurrent PROC. These results, presented in March 2026, establish GR antagonism as a promising therapeutic strategy and a potential new treatment paradigm in this setting. In this evidence-based review, we summarize the biological basis of GR signaling in ovarian cancer, evaluate the clinical evidence supporting SGRAs, and discuss key challenges for implementation, including biomarker development, safety considerations, and rational combination strategies with chemotherapy, PARP inhibitors, and immunotherapy. We also highlight critical considerations for future clinical trial design to optimize the integration of GR-targeted therapies into the management of ovarian cancer.

## 1 Introduction

OC, particularly high-grade serous ovarian cancer (HGSOC), is predominantly diagnosed at an advanced stage. Despite initial therapeutic efficacy, most patients experience recurrence and ultimately develop platinum resistance, leading to a worse prognosis (1). Consequently, the development of novel, effective therapies for PROC represents an urgent clinical need. In recent years, stress response pathways within the tumor microenvironment (TME), specifically the hypothalamic-pituitary-adrenal (HPA) axis-associated glucocorticoid signaling pathway, have emerged as key drivers of tumor progression and immune evasion (2, 3). Consistent with this, the GR is highly expressed in various solid tumors, including triple negative breast, prostate, cervical, and OCs, where its activation directly promotes tumor cell proliferation, survival, and invasion, while suppressing anti-tumor immune responses (4, 5). Building on this understanding, SGRAs have emerged as a therapeutic strategy to block this pro-tumorigenic pathway, with the aim of reversing chemotherapy resistance and remodeling the immunosuppressive TME (6). This review focuses on the therapeutic potential of SGRAs in OC, with particular emphasis on emerging clinical advances such as the Phase III ROSELLA trial.

## 2 The glucocorticoid receptor

The GR, encoded by the *NR3C1* gene, is a ubiquitously expressed, ligand-activated transcription factor that serves as the primary mediator for the multiple effects of glucocorticoid hormones (7). These hormones, the terminal products of HPA axis, are critical for maintaining metabolic homeostasis, modulating immune responses, regulating the body’s stress (8). The traditional view was that glucocorticoids act solely through the genomic pathway—the hormone binds to the GR, then moves into the nucleus and modulate gene transcription. However, emerging evidence indicate that GR signaling is more complex than initially appreciated. Research has revealed not only functionally distinct GR isoforms from alternative splicing and translation initiation, but also rapid non-genomic effects at the plasma membrane and intricate epigenetic layers that control GR expression and activity (9–11).

### 2.1 The role of GR signaling in the development and progression of OC

In ovarian physiology, GR signaling is activated by human chorionic gonadotropin (hCG), leading to the upregulation of key genes like *HSD11B1*, *NR3C1*, *FKBP5*, and *FKBP4* in periovulatory follicles and granulosa/lutein cells, highlighting its role in steroidogenesis and the ovulatory cascade (12). Beyond its classical genomic actions, GR can also engage in rapid non-genomic signaling pathways. Its expression is not consistently prognostic across all cancers, but meta-analyses indicate that high GR expression is specifically associated with an increased risk of progression in gynecological cancers, including OC (13, 14). This suggests that both canonical and non-canonical GR pathways may be exploited in OC.

### 2.2 GR signaling in OC cell proliferation, apoptosis resistance and epithelial-mesenchymal transition

GR activation plays a complex role in OC, physiological levels may suppress growth, while pathological or stress-induced signaling promotes tumorigenic features. A key mechanism involves GR-mediated resistance to apoptosis is that cortisol can blunt paclitaxel-induced cell death, but GR antagonists like relacorilant can reverse this effect and restore chemo sensitivity (15). Furthermore, psychological stress activates GR, which directly promotes epithelial-mesenchymal transition (EMT) and metastasis by transcriptionally upregulating Nuclear Protein 1 (NUPR1). NUPR1 subsequently increases the expression of SNAI2 (SLUG), a master regulator of EMT, establishing a direct molecular link between GR signaling and enhanced invasive potential in OC (16).

### 2.3 Activation of the GR pathway shapes an immunosuppressive TME in OC, facilitating immune escape

Chronic stress, glucocorticoids like corticosterone, drives polymorphonuclear myeloid-derived suppressor cells (PMN-MDSCs) into the TME, where they suppress anti-tumor immunity and promote cancer progression (17). Evolutionary analyses of cancer genomes indicate that mutations in the GR regulatory network tend to appear in the early stage of tumor development. That likely primes the TME for immune evasion and tumor growth from the outset (18). This immunosuppression driven by stress hormones helps tumors grow and puts up a major roadblock to effective immunotherapy. GR activation can also promote immune evasion potentially through interactions with regulatory T cells (Tregs) (19, 20), as demonstrated in cervical cancer studies: GR expression correlates with FoxP3 (a Treg marker) and is tied to worse prognosis and platinum resistance (5). This immunosuppressive role positions GR as a key modulator of the tumor-immune interface.

### 2.4 GR signaling mediates chemotherapy resistance in OC

High GR expression has been clinically associated with poor prognosis and impaired chemotherapy effectiveness, as GR activation can induce pro-survival pathways and cell differentiation (4). Critically, GR activation has been shown to impair chemotherapy effectiveness by modulating apoptotic pathways, driving treatment resistance (21, 22). Cisplatin can bind to GR and activate it, leading to higher levels of resistance factors like MAST1. This reactivates MAPK signaling and contributes to treatment failure. This mechanism is supported by clinical observations of GR nuclear translocation and MAST1 upregulation in patient tumors following platinum treatment, correlating with resistance (23).

**FIGURE 1:**
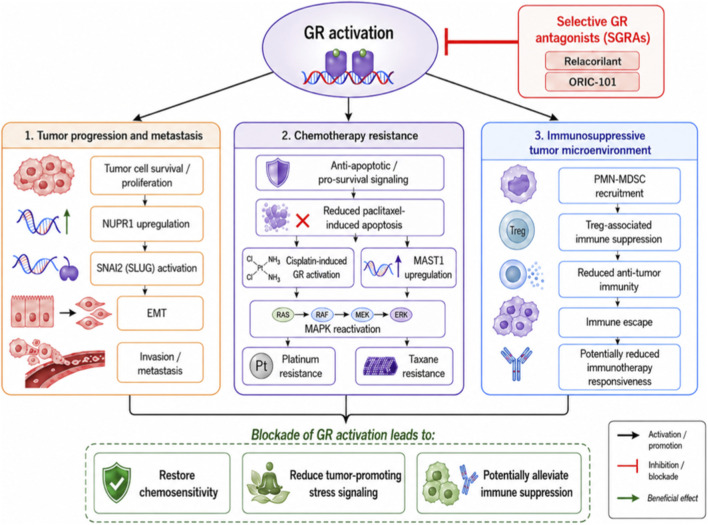
Proposed roles of glucocorticoid receptor signaling in ovarian cancer progression, chemotherapy resistance, and immune suppression. Activation of the glucocorticoid receptor (GR) promotes tumor cell survival and proliferation and may facilitate epithelial–mesenchymal transition (EMT), invasion, and metastasis through upregulation of NUPR1 and SNAI2/SLUG. GR signaling also contributes to chemotherapy resistance by activating anti-apoptotic and pro-survival pathways, reducing paclitaxel-induced apoptosis, and promoting cisplatin-associated MAST1 upregulation and MAPK pathway reactivation, thereby supporting platinum and taxane resistance. In the tumor microenvironment, GR signaling may promote recruitment of polymorphonuclear myeloid-derived suppressor cells (PMN-MDSCs), regulatory T cell-associated immune suppression, reduced antitumor immunity, and immune escape. Selective GR antagonists (SGRAs), including relacorilant and ORIC-101, inhibit GR signaling and may restore chemosensitivity, reduce tumor-promoting stress signaling, and alleviate GR-mediated immune suppression. Black arrows indicate activation or promotion, red lines indicate inhibition or blockade, and green arrows indicate potential beneficial effects.

Therefore, selectively inhibiting GR offers a dual strategy: to directly sensitize cancer cells to chemotherapy-induced apoptosis and indirectly enhancing anti-tumor immunity through relief of GR-mediated immunosuppression (24). SGRAs development and the critical clinical evidence—most notably from the ROSELLA trial—are shaping this new therapeutic strategy for PROC, as discussed in the following sections. The major roles of GR signaling in ovarian cancer progression, chemotherapy resistance, and immune suppression, together with the potential therapeutic effects of selective GR antagonists, are summarized in Figure 1. Herein, we summarize the registered clinical trials targeting GR in OC as of 10 April 2026, from ClinicalTrials.gov, as shown in Table 1.

**TABLE 1:** Registered clinical trials targeting GR in OC.

NCT	Intervention	Condition	Phase	Patients number	Status	Results
00459290	Mifepristone	Recurrent or persistent EOC, fallopian tube, primary peritoneal carcinoma	II	24	Completed	PFS at 6 months: 13.6% Objective tumor response: 4.5% AE (grade 3 or higher): 27.3%
02014337	Mifepristone + Eribulin	Solid tumors (including recurrent EOC)	I	37	Completed	N/A
02046421	Mifepritone, Gemcitabine, Carboplatin	Recurrent or persistent EOC, fallopian tube, primary peritoneal carcinoma	I	31	Completed	N/A
02762981	RELA + Nab-p	Solid tumor	I/II	85	Completed	Objective response rate: 13% (phase I), 10.5% (phase II)
03928314	ORIC-101	Solid tumor	I	83	Terminated	N/A
03776812 (25)	RELA + Nab-p	PROC	II	Arm A (intermittent RELA + Nab-p): 60 Arm B (continuous RELA + Nab-p): 58 Arm C(Nab-p): 60	Completed	mPFS: A vs. C: 5.6 months vs. 3.8 months B: 5.3 months mOS: A: 13.9 months B: 11.3 months C: 12.2 months
05257408 (26)	RELA + Nab-p	PROC	III	Arm A (RELA + Nab-p): 188 Arm B (Nab-p): 193	Ongoing	mPFS A: 6.54 months B: 5.52 months mOS A: 15.97 months B: 11.50 months
06906341	RELA + Nab-p +Bevacizumab	Gynecological cancers (including OC)	II	270	Recruiting	N/A

Abbreviation: RELA, relacorilant; Nab-p, Nab-paclitaxel.

## 3 SGRAs: A novel therapeutic strategy

SGRAs block the pro-tumorigenic and chemoresistance-promoting signals that come from GR activation. By antagonizing GR, they reverse chemotherapy resistance and reshape the immunosuppressive TME, thereby positioning SGRAs as a promising option for resistant diseases like PROC (4). The development of SGRAs, such as relacorilant, marks a significant step in translating this biological understanding into clinical intervention.

### 3.1 Drug development and mechanism of action of SGRAs

First-generation GR antagonists like mifepristone (RU486) have provided foundational insights but possess significant limitations due to their non-selective nature. Mifepristone acts as a dual antagonist for both the progesterone receptor (PR) and the GR, which can lead to off-target hormonal effects that complicate its therapeutic use, particularly in oncology where precise pathway modulation is desired (12). Preclinical studies show mifepristone can reduce cortisol and progesterone production in human granulosa/lutein cells and may help overcome resistance to BRAF inhibitors in melanoma, its clinical utility is constrained by this lack of specificity (27). These limitations underscored the need for SGRAs without interfering with other steroid pathways, to minimize side effects and let us more cleanly probe GR’s specific role in disease and treatment resistance.

Newer GR antagonists like relacorilant (CORT125134) and ORIC-101 were developed to overcome the drawbacks of non-selective drugs. These compounds specifically and potently block GR signaling without interfering with PR or other steroid receptors. CORT125134, for example, selectively blocks hCG-induced cortisol and progesterone production in cellular models (12, 28).

The high selectivity results in an improved therapeutic window due to minimized off-target effects. The drugs are optimized to potently downregulate GR signaling, which clinical pharmacodynamic data has been confirmed in clinical pharmacodynamic analyses (4), positioning these agents as tools to specifically interrogate and counteract GR-mediated mechanisms of chemotherapy resistance.

### 3.2 SGRAs in OC: an in-depth analysis of the ROSELLA trial

Preclinical studies show that SGRAs reverse chemotherapy resistance and may also modulate the TME. In OC xenograft models, GR activation impairs chemotherapy effectiveness such as taxanes by turning on anti-apoptotic pathways, and GR expression is linked to poor prognosis (4). SGRAs like relacorilant and ORIC-101 were therefore developed to disrupt this adaptive signaling and re-sensitize tumors to chemotherapies. SGRA–paclitaxel combinations induced significant tumor regression even in taxane-resistant settings (29, 30). Collectively, these findings provide a strong mechanistic rationale for translating GR blockade into the clinical setting, exemplified by the evaluation of relacorilant in combination with chemotherapy in the ROSELLA trial.

The ROSELLA trial was a pivotal, global, randomized, open-label, phase 3 study designed to evaluate the efficacy of relacorilant, a first-in-class SGRA, combined with nab-paclitaxel in PROC (26). The trial enrolled adult patients with confirmed PROC, primary peritoneal, or fallopian tube cancer (high-grade serous, endometrioid, or carcinosarcoma with a ≥30% epithelial component) who had received up to three prior lines of therapy, must include prior bevacizumab, and had measurable disease per RECIST v1.1 with an ECOG performance status of 0 or 1. Patients were randomized 1:1 to receive either relacorilant (150 mg orally on days −1, 1, and 2 of each cycle) plus a lower dose of nab-paclitaxel (80 mg/m^2^) or a higher dose of nab-paclitaxel monotherapy (100 mg/m^2^), both administered intravenously on days 1, 8, and 15 of a 28-day cycle. The reduced nab-paclitaxel dose (80 mg/m^2^) in the combination arm was selected based on phase I findings that relacorilant inhibits CYP3A4-mediated paclitaxel metabolism, resulting in systemic exposures equivalent to nab-paclitaxel 100 mg/m^2^ alone (31), and was validated in the phase II study which showed improved efficacy without additional toxicity (25).

The dual primary endpoints of the ROSELLA trial were progression-free survival (PFS) as assessed by blinded independent central review (BICR) and overall survival (OS). The interim analysis demonstrated a statistically significant improvement in PFS for the combination arm, with a median PFS of 6.54 months compared to 5.52 months for nab-paclitaxel monotherapy (HR 0.70; 95% CI 0.54–0.91; *p* = 0.0076). Moreover, at 76% data maturity and with a median follow-up of 24.8 months (95% CI 23.6–25.7), the relacorilant combination group showed a 4.1-month improvement in median OS compared with nab-paclitaxel alone (16.0 months [95% CI 13.0–18.3] vs. 11.9 months [10.0–13.8], respectively) (32). These findings support the clinical efficacy of the combination. Regarding safety, adverse events were generally comparable between groups after adjustment for nab-paclitaxel exposure. Relacorilant showed no new safety signals, although toxicity was higher in the combination arm.

The preliminary results from the ROSELLA trial represent a significant advance in the treatment of PROC, a setting with limited effective options. The combination of relacorilant and nab-paclitaxel demonstrates that targeting GR signaling can enhance chemotherapy efficacy in patients, translating a biological concept into clinical benefit (4). Notably, the intermittent dosing strategy appears sufficient to mitigate cortisol-mediated pro-survival signaling during chemotherapy exposure while maintaining an acceptable safety profile, supporting a pharmacologically rational approach to GR modulation.

An ongoing analysis from the ROSELLA trial aims to explore biomarkers that identify which patients benefit most from SGRAs. Preclinical data indicates that high GR expression is associated with poor prognostic features in OC and may serve as a predictive biomarker for response to relacorilant-based therapy. In addition, the crosstalk between GR signaling and other oncogenic pathways, such as inflammatory pathways (e.g., NF-κB, JAK-STAT) and estrogen receptor signaling, may reveal molecular subtypes with heightened dependence on GR-mediated survival signaling (33). Physiologic evidence from normal ovarian biology, where hCG-driven GR activation regulates steroidogenesis and the ovulatory cascade, supports the notion that hormonally driven tumors may be particularly susceptible to GR antagonism (12). Collectively, these observations highlight the need for robust biomarker strategies to enable patient stratification and optimize the depth and durability of response to relacorilant-based combinations.

## 4 Current clinical research challenges and future directions

### 4.1 Patient selection and the challenge of identifying predictive biomarkers for efficacy

A major challenge in the clinical development of GR-targeted therapy in OC is the identification of reliable biomarkers to guide patient selection. While elevated GR expression has been associated with poor prognosis, its utility as a predictive marker for response to SGRAs remains uncertain, as expression alone may not fully capture functional pathway dependence (4). Notably, The ROSELLA trial did not use biomarker-based selection, yet it showed overall efficacy in an all-comer population. However emerging evidence suggests that response to SGRAs is likely shaped by broader tumor biology, including BRCA status, homologous recombination proficiency, and co-activation of parallel survival pathways. These considerations highlight the heterogeneity of GR signaling across OC subtypes and underscores the need for integrative biomarker strategies that incorporate both pathway activity and tumor genomic context (34).

### 4.2 Safety management: monitoring potential impact on the HPA axis

Targeting GR signaling introduces unique safety considerations, particularly with respect to the HPA axis. While exogenous glucocorticoids are widely used alongside other drugs in oncology, their chronic administration is associated with significant toxicity, including HPA axis suppression, which causes treatment-related morbidity (35). In contrast, SGRAs like relacorilant aim to dissociate therapeutic effects from adverse glucocorticoid effects. Early-phase trials combining SGRAs with other therapies, such as enzalutamide in prostate cancer, have reported that the combinations are generally well-tolerated, suggesting a potentially improved safety profile regarding steroid-related toxicities (36). However, given the central role of GR in endocrine homeostasis, monitoring of HPA axis function remains essential, particularly with prolonged or combination use. Long-term data are limited, and further studies are needed to define risks such as adrenal insufficiency and optimal monitoring strategies.

### 4.3 Exploration of combination strategies: synergistic effects with chemotherapy, PARP inhibitors, and immunotherapy

Future strategies for GR-targeted therapy in OC focus on rational combination approaches to overcome resistance and improve outcomes. Recently, agents such as the folate receptor-targeted ADC mirvetuximab soravtansine (MIRASOL trial) and the immune checkpoint inhibitor pembrolizumab (KEYNOTE-B96 trial) have been successively approved by the FDA for the treatment of PROC. Notably, the phase 3 ROSELLA trial, presented on 25 March 2026, demonstrated that adding the SGRA relacorilant to nab-paclitaxel significantly improved PFS and OS in PROC, establishing a mechanistically informed backbone for future combination strategies (37). Although these approvals offer additional treatment options, the optimal sequencing of SGRAs, ADCs, and immunotherapies remains undefined. Key questions include whether these agents should be prioritized based on biomarker status, prior treatment exposure, toxicity profiles, or mechanism of resistance, and whether sequential or combination strategies will provide greater clinical benefit. Future studies should therefore focus not only on developing new agents but also on defining rational treatment sequences to optimize outcomes for patients with PROC.

Beyond chemotherapy, other options such as anti-angiogenic agents also deserve exploration. In the platinum-resistant phase, most patients may have already received bevacizumab. Therefore, the potential benefit of combining anti-angiogenic agents with SGRA therapy must be weighed against the risk of resistance from prior exposure versus potential synergism. Looking back at the inclusion criteria of the ROSELLA trial, all enrolled patients had a prior history of bevacizumab. Meanwhile, in the ROSELLA trial, 3% of patients in the subsequent treatment setting received a chemotherapy plus bevacizumab regimen (38). This may be based on the fact that adding bevacizumab to chemotherapy can improve the objective response rate to approximately more 16% in PROC. Therefore, whether combining SGRAs with bevacizumab could further enhance efficacy is worth investigating, including ongoing gynecologic cancer clinical studies combining SGRAs with chemotherapy and bevacizumab (NCT:06906341).

Combining SGRAs with PARP inhibitors (PARPi) may enhance efficacy in tumors with homologous recombination proficiency (HRp), where PARPi monotherapy is less effective, though this requires further clinical validation (34). Furthermore, given that OC is often immunologically “cold”, targeting GR to modulate the TME and enhance responsiveness to immunotherapies such as immune checkpoint inhibitors is worth exploring (39). A deeper understanding of GR-mediated effects on immune cell populations will be critical to guide these combination therapies and optimize patient outcomes (40).

### 4.4 Design considerations and key success factors for future phase III clinical trials

Future clinical trials for SGRAs in PROC must address prior challenges to demonstrate clear clinical benefit, including the need for a precise and consistent definition of platinum resistance (41). The success of the ROSELLA trial underscores the importance of a rational, biologically grounded combination, pairing the SGRAs with a chemo drug whose resistance is mediated by GR signaling (26). Trial design should also incorporate patient-reported outcome (PRO) assessments as key secondary endpoints to complement traditional toxicity reports and capture effects on quality of life (42). Concurrently, biomarker development remains essential; correlative studies evaluating GR expression, pathway activity, and TME features will be critical for patient stratification, enabling personalized therapy and improving the likelihood of trial success and clinical impact (43). Several biomarker strategies warrant further investigation, including GR expression levels, GR transcriptional signatures, and cortisol dynamics. Among these, GR transcriptional signatures may be particularly informative because they reflect functional pathway activity rather than static expression alone. Cortisol dynamics remain less defined for patient selection, although peripheral cortisol levels may have values as pharmacodynamic markers (44).

## 5 Conclusion

The emergence of SGRAs marks a significant and promising advance in the treatment of OC, particularly for the platinum-resistant population with limited options. Translating mechanistic insights into targeted clinical application, foundational research has established the central role of GR signaling in tumor progression, chemotherapy resistance, and the immunosuppressive TME, providing a strong rationale for selective inhibition. Agents such as relacorilant, with high GR selectivity, balance therapeutic efficacy with improved safety compared to earlier non-selective antagonists.

The phase 3 ROSELLA trial validated the strategic approach and provides a crucial proof-of-concept for future strategies. Moving forward, the clinical impact of SGRAs will depend on the identification of predictive biomarkers to enable precision patient selection, optimization of dosing and scheduling, and rational combination strategies with chemotherapy, PARP inhibitors, or immunotherapies. With continued mechanistic and correlative studies, SGRAs have the potential to become an integral component of OC therapy, offering a novel mechanism to improve patient outcomes.
